# pH-Responsive Emission of Novel Water-Soluble Polymeric Iridium(III) Complexes

**DOI:** 10.3390/nano12060927

**Published:** 2022-03-11

**Authors:** Dafnianna Tsakaraki, Aikaterini K. Andreopoulou, Georgios Bokias

**Affiliations:** 1 Department of Chemistry, University of Patras, GR 26504 Patras, Greece; dafnianna@windowslive.com (D.T.); andreopo@upatras.gr (A.K.A.); 2 FORTH/ICE-HT, Stadiou Street, P.O. Box 1414, GR 26504 Rio-Patras, Greece

**Keywords:** Iridium polymeric complexes, water-soluble copolymers, water-soluble metallocomplexes, pH-sensors, quinoline-pyridine ligand, photoluminescence

## Abstract

The synthesis and characterization of water-soluble copolymers containing *N,N*-dimethylacrylamide (DMAM) and a vinylic monomer containing an Iridium(III), Ir(III), complex substituted with the quinoline-based unit 2-(pyridin-2-ylo)-6-styrene-4-phenylquinoline (VQPy) as ligand are reported. These copolymers were prepared through pre- or post-polymerization complexation of Ir(III) with the VQPy units. The first methodology led to copolymer **P1** having fully complexed VQPy units, whereas the latter methodology allowed the preparation of terpolymers containing free and Ir(III)-complexed VQPy units (copolymer **P2**). The optical properties of the copolymers were studied in detail through UV-Vis and photoluminescence spectroscopy in aqueous solution. It is shown that the metal-to-ligand charge transfer (ΜLCT) emission is prevailing in the case of **P1**, regardless of pH. In contrast, in the case of terpolymer **P2** the MLCT emission of the Ir(III) complex is combined with the pH-responsive emission of free VQPy units, leading to characteristic pH-responsive color changes under UV illumination in the acidic pH region.

## 1. Introduction

Transition-metal complexes are well-known for their ability to achieve high-efficiency phosphorescence because they can harvest both singlet and triplet excitons. Motivated by this property, cyclometalated complexes based on metal ions such as Ru(II), Rh(I), Pt(II), and Ir(III) have been largely investigated [1,2,3,4]. Among these, iridium(III) complexes have attracted great attention because of their special optical properties [5,6,7], namely the ability to harvest both singlet and triplet exciton, their high photoluminescence efficiency and high quantum yields, wide range of colors emitted etc. Thus, Ir(III) complexes are promising materials for a wide variety of applications, including among others, organic light emitting diodes (OLEDs) [8,9,10,11], bioprobes [12,13,14,15,16,17,18], catalysts [19,20,21,22,23], optical chemical sensors [24,25,26,27,28,29,30,31].

Ιr(III) complexes are most commonly soluble in organic solvents rather than in aqueous solutions. In contrast, the development of water-soluble luminescent Ir(III) complexes is valuable for biological and biochemical applications such as bioprobes for cell imaging or their use as indicators to determine parameters such as pH, temperature, or oxygen. To this end, the ligands may be functionalized with hydrophilic groups [13,14,32,33,34,35,36,37] or the counter ions of cationic cyclometalated Ir(III) complexes may be adequately chosen to sustain water-solubility [14,38,39,40]. Alternatively, the covalent attachment of Ir(III) complexes onto a polymeric chain, using water-soluble polymers such as poly(N-isopropylacrylamide), poly(N-vinylpyrrolidone), and polyethyleneglycols, has been also applied [41,42,43,44,45,46,47,48,49,50].

The development of pH sensors is of great interest, since they are used in many different areas, such as environmental monitoring, biology, and food industry. For our current approach, we take advantage of our experience about the functionalization of polymeric or carbon materials with pH-sensitive luminescent units based on quinoline derivatives. Due to its weak basic character, the protonation/deprotonation of the quinoline group results in the change of the emitted color from blue to green upon decreasing pH [51,52,53,54]. The vinyl-functionalization of quinoline derivatives and subsequent copolymerization allowed us to tune these pH-responsive emission properties [55] or to combine them with additional functionalities, such as thermosensitivity [56], selective surfactant sensing [57], as well as encapsulation abilities for hydrophobic magnetic nanoparticles [58].

**Scheme 1:**
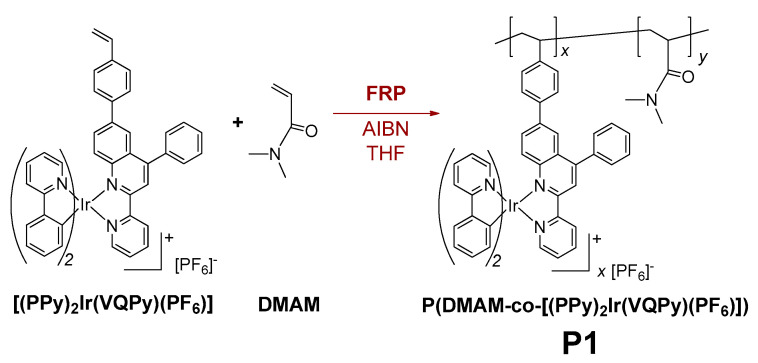
Synthesis of P(DMAM-co-[(PPy)_2_Ir(VQPy)(PF_6_)]) **P1**, through direct free radical polymerization of DMAM with [(PPy)_2_Ir(VQPy)(PF_6_)].

Recently, in our group, organo-soluble polymeric Ir(III) complexes were successfully prepared through “post-polymerization” or “pre-polymerization” complexation, aiming at OLED applications [59,60]. Our target in the present work is the combination of the aforementioned pH-responsive properties of quinoline derivatives with the optical properties of Ir(III) complexes into a water-soluble polymeric nanostructure. To this end, a vinylic monomer containing a pyridine-quinoline-based ligand [61], namely 2-(pyridin-2-ylo)-6-styrene-4-phenylquinoline (VQPy), was explored as the polymerizable ligand of Ir(III) ions. Based on this ligand, highly luminescent water-soluble polymeric materials have been developed by introducing the iridium(III) complex (PPy)_2_Ir(VQPy) (PPy: phenylpyridine) as signaling units along a nonionic hydrophilic N,N-dimethylacrylamide, DMAM, chain (see Scheme 1), through free radical polymerization in organic solvents. While “pre-polymerization” complexation assures that all VQPy units are complexed, “post-polymerization” complexation offers the possibility to control the fraction of complexed VQPy units and to tune thus the optical properties of this metallocomplex nanomaterial.

## 2. Materials and Methods

### 2.1. Materials

The monomer 2-(pyridin-2-ylo)-6-styrene-4-phenylquinoline) (VQPy) was synthesized according to literature procedures [62], through a Friedländer reaction followed by a Suzuki cross-coupling reaction. The monomer Ν,Ν-dimethylacrylamide (DMAM), the reagent ammonium hexafluorophosphate (NH_4_PF_6_), the solvent chloroform (CHCl_3_) were purchased from Sigma-Aldrich (Sigma-Aldrich Chemie GmbH, Taufkirchen, Germany). The reagents 2-phenylpyridine (PPy), IrCl_3_*H_2_O and the solvent 2-ethoxyethanol were purchased from Alfa Aesar (Thermo Fisher (Kandel) GmbH Postfach, Karlsruhe, Germany). The initiator α’–α’ azobisisobutironitrile (AIBN), the reagents for the preparation of the buffer solutions, trisodium citrate dihydrate, and hydrochloric acid, the solvents tetrahydrofuran (THF), diethyl ether (Et_2_O), and methanol were purchased from Merck (Merck KGaA, Darmstadt, Germany). Ultra-pure water was obtained by means of a SG Water laboratory unit.

### 2.2. Instrumentation

^1^H-NMR spectra were obtained on a Bruker Advance DPX 400.13 MHz spectrometer (Bruker BioSpin GmbH, Magnet Division, Karlsruhe, Germany) at 25 °C using deuterated CDCl_3_ or *d6*-DMSO. Chemical shifts (δ) are reported in units, parts per million (ppm) downfield from TMS.

Attenuated total reflectance Fourier transform infrared spectra (ATR-FTIR) were recorded on a “Bruker Optics’ Alpha-P Diamond ATR Spectrometer of Bruker Optics GmbH” (Ettlingen, Germany).

A single-quadrupole Quattro micro mass spectrometer (ACQUITY SQ Detector) equipped with an electrospray ionization (ESI) interface was used for analytical detection. ESI-MS was operated in positive mode under the following operating parameters: capillary voltage 3.5 kV, cone voltage 30 V, source temperature 140 °C, desolvation temperature 250 °C, desolvation gas (nitrogen) 500 L/hour, and cone gas (nitrogen) 50 L/h. All data were acquired and processed using Masslynx 4.1 software (Waters Corp., Milford, MA, USA).

Size-exclusion chromatography (SEC) measurements were carried out using a Polymer Lab chromatographer (Agilent Technologies, Santa Clara, CA, USA) equipped with two PLgel 5 μm mixed columns and a UV detector using CHCl_3_ as the eluent at a flow rate of 1 mL min^−1^ at 25 °C; and calibrated versus polystyrene standards.

The UV–Vis spectra were recorded using a Hitachi-Science and Technology-U-1800 UV–Vis Spectrophotometer in the 190–800 nm wavelength range (Hitachi High-Technologies Europe GmbH, Mannheim, Germany).

Photoluminescence (PL) spectra were recorded using a Perkin–Elmer LS45 luminescence spectrometer (Waltham, MA, USA), using quartz glass cuvettes (optical path = 1 cm). For the photoluminescence study, the excitation and emission slits were 10 nm, while the excitation wavelengths were 350 nm or 400 nm.

### 2.3. Synthesis of Compounds

**[(PPy)_2_Ir(VQPy)(PF_6_)] complex. ** The complex was synthesized in two steps [63,64]. First, the chloro-bridged dinuclear cyclometalated iridium(III) [(PPy)_2_IrCl_2_]_2_ was synthesized: 2-phenylpyridine (PPy) (127 μL, 0.880 mmol) was mixed with IrCl_3_*H_2_O (0.106 g, 0.360 mmol) in a mixture of 2-ethoxyethanol and H_2_O (3/1 *v*/*v*) to reach a concentration in IrCl_3_*H_2_O of ~0.04 M. The mixture was degassed by multiple vacuum and N_2_ purging cycles and was heated in an oil bath set at 120 °C for 24 h under N_2_. The yellow precipitate formed during the reaction was filtered, washed with hot methanol, and dried under vacuum at 40 °C overnight. In the second step, VQPy (0.045 g, 0.120 mmol) and 2.3 mL 2-ethoxyethanol were added to 0.050 g (0.050 mmol) of the yellow precipitate, to reach again a concentration of ~0.04 M. The suspension was heated in an oil bath set at 130 °C for 24 h under N_2_, leading to a red solution_._ The reaction mixture was cooled to room temperature and was extracted several times with Et_2_O and water. After the addition of a solution of NH_4_PF_6_ (10 eq in respect to the dinuclear [(PPy)_2_IrCl]_2_ complex) in the aqueous phase, a red solid was precipitated. The suspension was cooled to 0 °C for 1 h, filtered and washed with H_2_O. The red solid was dried under vacuum at 60 °C overnight.

**Scheme 2:**
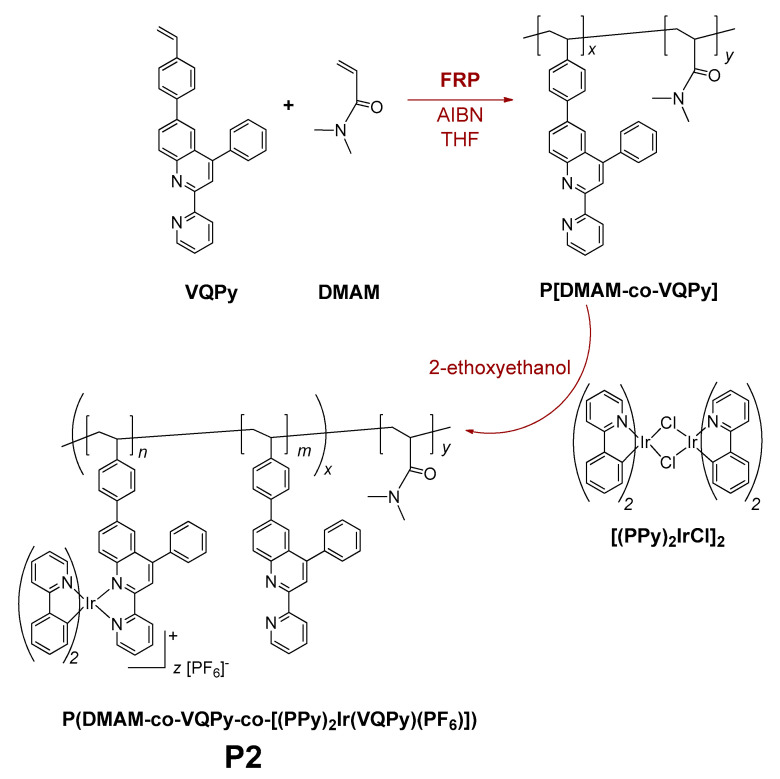
Synthesis of P(DMAM-co-VQPy-co-[(PPy)_2_Ir(VQPy)(PF_6_)]) **P2**, through reaction between the dinuclear [(PPy)_2_IrCl_2_]_2_ complex and the copolymer P(DMAM-co-VQPy).

**Synthesis of P(DMAM-co-[(PPy)_2_Ir(VQPy)(PF_6_)]) copolymers.** Two different synthetic procedures were followed as depicted in Scheme 1 and Scheme 2. Both of them were based on the free radical copolymerization of DMAM with VQPy, either complexed with Ir(III) (Scheme 1) or free (Scheme 2).

**Direct copolymerization of DMAM with [(PPy)_2_Ir(VQPy)(PF_6_)] (Scheme 1).** THF was degassed by bubbling with N_2_ to remove the dissolved oxygen. Τhe monomers DMAM (0.4458 g, 4.5 mmol) and [(PPy)_2_Ir(VQPy)(PF_6_)] (0.0200 g, 0.023 mmol), as well as the initiator AIBN (2% mol over the total monomers quantity), were added in the solvent and the mixture was stirred at 70 °C for 2 days. The reaction mixture was added in Et_2_O under stirring, leading to the precipitation of a slightly orange solid. The mixture was filtered, and the resulting product **P1** was washed several times with cold Et_2_O and recovered after drying in vacuum at 40 °C. Mn = 10.000 Da, Polydispersity Index = 2.1.

**Copolymerization of DMAM with VQPy and subsequent introduction of Ir(III) complex (Scheme 2).** According to the second methodology, the synthesis of the P(DMAM-co-VQPy) through free radical polymerization of DMAM with VQPy preceded the complexation reaction. The monomers DMAM (1.5 mL 14.800 mmol) and VQPy (0.300 g 0.780 mmol) and the initiator AIBN (0.0513 g, 0.3124 mmol) were added in freshly distilled and deoxygenated THF (18 mL) and the mixture was stirred at 70 °C for 2 days. The polymer was precipitated in Et_2_O and the white solid was filtered, washed with cold Et_2_O, and dried under vacuum. Mn = 15.000 Da. Poldispersity Index = 1.8.

In the second step, the copolymer P(DMAM-co-VQPy) (0.4595 g, 0.2300 meq of VQPy,) was mixed with the chloro-bridge dinuclear [(PPy)_2_IrCl]_2_ complex (0.050 g, 0.093 mmol of Ir) in 2-ethoxyethanol (2.3 mL, the ethoxyethanol/Ir molar ratio was about 4) at 130 °C for 24 h under N_2_. The reaction mixture was cooled to room temperature and 3 mL deionized H_2_O was added. Then the biphasic solution was extracted with H_2_O and Et_2_O and the collected water phase was heated at 70 °C for 30 min. Then ΝH_4_PF_6_ was added (10 eq in respect to the dinuclear [(PPy)_2_IrCl]_2_ complex) without however leading to the precipitation of the polymeric complex. Thus, the polymer **P2** was purified through dialysis (membrane, MWCO: 12.000 Da) and recovered through freeze-drying. Mn = 12.000 Da, Polydispersity Index = 1.5.

## 3. Results

### 3.1. Synthesis and Characterization of Compounds

**Figure 1:**
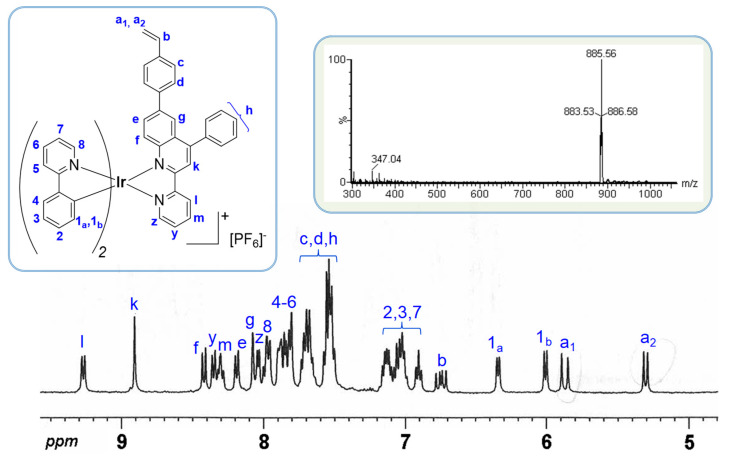
^1^H NMR spectrum of complex [(PPy)_2_Ir(VQPy)(PF_6_)] in DMSO-*d6*. The inset shows the respective MS-ESI spectrum.

The vinylic complex [(PPy)_2_Ir(VQPy)(PF_6_)] was synthesized in two steps, involving initially the preparation of the chloro-bridged dinuclear cyclometalated iridium(III) complex [(PPy)_2_IrCl_2_]_2_ and the subsequent reaction of this complex with the vinylic monomer VQPy. The final complex was characterized through ^1^H NMR and MS-ESI. The detailed attribution of the peaks observed in the ^1^H NMR spectrum is shown in Figure 1. As seen, the characteristic peaks of the vinylic bond and the peaks attributed to the aromatic groups of the complex are clearly detected. In addition, the verification of the synthesis was also based on the determination of the molecular weight of the complex through MS-ESI (Figure 1, inset). The main product of the sample with a molecular weight of 885.56 g/mol is revealed in the MS-ESI spectrum. Having in mind that the expected molecular weight of the complex is 886.62 g/mol, this suggests that the desired complex has been successfully synthesized.

For the incorporation of this complex into the water-soluble poly(N,N-dimethylacrylamide) chain, two alternative methodologies were followed. According to the first one, namely pre-polymerization Ir(III) complexation, the target copolymer P(DMAM-co-[(PPy)_2_Ir(VQPy)(PF_6_)]) was synthesized via free radical copolymerization of the monomer DMAM with the vinyl-modified complex [(PPy)_2_Ir(VQPy)(PF_6_)] (Scheme 1, product **P1**). The feed molar content of the Ir(III) complex was adjusted at 0.5 mol% for this product.

The second methodology, namely the post-polymerization Ir(III) complexation, is illustrated in Scheme 2. For this route, as a first step, the copolymer P(DMAM-co-VQPy) was initially synthesized through free radical copolymerization in an organic solvent. The molar feed composition of VQPy units was adjusted at ~5mol%. The second step was the complexation of VQPy units of the copolymer with Ir using the chloro-bridged dinuclear complex [(PPy)_2_IrCl]_2._ The molar ratio of [(PPy)_2_IrCl]_2_ over VQPy units of the copolymer (based on feed composition) was 0.2. As a consequence, the final product is expected to be a terpolymer, namely P(DMAM-co-VQPy-co-[(PPy)_2_Ir(VQPy)(PF_6_)]) (product **P2)**, containing comparable contents of complexed and free VQPy units.

**Figure 2:**
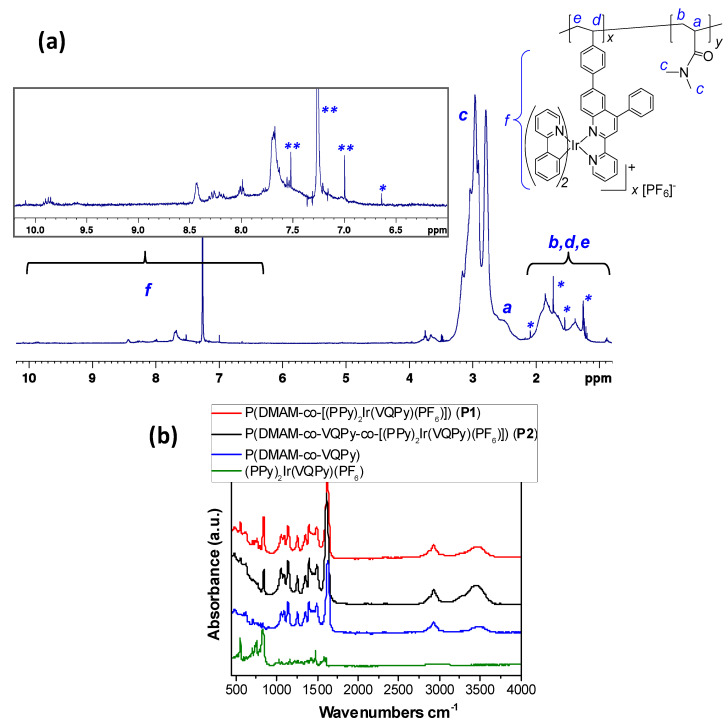
(**a**) ^1^H NMR spectrum in CDCl_3_ of the copolymer P(DMAM-co-[(PPy)_2_Ir(VQPy)(PF_6_)]) **P1** and (**b**) ATR spectra of the copolymers P(DMAM-co-[(PPy)_2_Ir(VQPy)(PF_6_)]) **P1,** P(DMAM-co-VQPy-co-[(PPy)_2_Ir(VQPy)(PF_6_)]) **P2** and P(DMAM-co-VQPy) and of the monomer complex [(PPy)_2_Ir(VQPy)(PF_6_)]. In (**a**) peaks denoted with ** asterisks are the residual CHCl_3_ of CDCl_3_ and its symmetric ^13^C satellites peaks, while peaks denoted with * asterisk are residual solvent traces.

The products **P1** and **P2** were characterized by ^1^H NMR and ATR-IR. As seen in Figure 2, where the ^1^H NMR spectrum of product **P1** is shown as an example, weak peaks are observed in the region 7–9 ppm, as a consequence of the aromatic content owing to the [(PPy)_2_Ir(VQPy)(PF_6_)] units of this copolymer. In addition, the peaks in the 2.5–3 ppm region are attributed to the methyl groups of the DMAM units, while the methylene and methine protons of the polymeric backbone are found in the 1–2.5 ppm region.

The AΤR spectra of products **P1** and **P2** are compared in Figure 2b with the respective spectra of the copolymer P(DMAM-co-VQPy) and the vinylic Ir(III) complex [(PPy)_2_Ir(VQPy)(PF_6_)]. In the spectrum of the monomer complex, the peaks at ~840 cm^−1^ and ~550 cm^−1^ are attributed to the existence of hexafluorophosphate. As it can be observed, the same peaks appear at the spectrum of the copolymer P(DMAM-co-[(PPy)_2_Ir(VQPy)(PF_6_)]) but do not exist at the spectrum of the copolymer P(DMAM-co-VQPy). Finally, the peak at the 1610 cm^−1^ is attributed to quinoline VQPy double bond (C=C and C=N) of the aromatic rings. The peaks at ~2900 cm^−1^ of the copolymers’ spectra are attributed to the main polymer backbone C-H bonds.

### 3.2. Optical Properties

**Figure 3:**
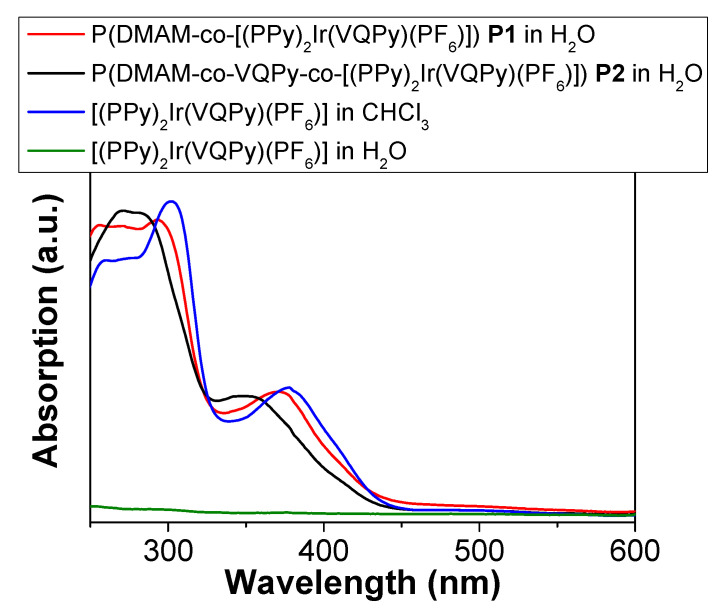
UV-Vis absorption spectra of the copolymers P(DMAM-co-[(PPy)_2_Ir(VQPy)(PF_6_)]) **P1**, P(DMAM-co-VQPy-co-[(PPy)_2_Ir(VQPy)(PF_6_)]) **P2**, in aqueous solution and of the complex [(PPy)_2_Ir(VQPy)(PF_6_)] in CHCl_3_ and in water.

The optical properties of the Ir(III)-containing copolymer obtained through both methodologies were investigated in aqueous solution. It is worthy to note that the initial complex [(PPy)_2_Ir(VQPy)(PF_6_)] is practically insoluble in water and thus, no UV-Vis absorbance is monitored when trying to study the aqueous dispersion of this material, as shown in Figure 3. In contrast, it is readily soluble in chloroform, exhibiting the characteristic properties of such Ir(III) complexes. In particular, the strong absorption band at ~300 nm, closely resembling the absorption spectrum of the free ligand VQPy [54], is attributed to the π-π* transition of the quinoline group. In addition, the complex presents weaker absorption bands in the range of 350–450 nm, which are attributed to the metal-to-ligand charge transfer (MLCT) transitions. More precisely, the absorption band at 350–400 nm is assigned to singlet metal-to-ligand charge transfer (^1^MLCT) transitions, whereas the weaker absorption area above 400 nm is attributed to triplet metal-to-ligand charge transfer (^3^MLCT) transitions [65,66]. The same absorption bands are also observed in the cases of the two copolymers. However, it should be stressed that now this behavior is monitored in aqueous solution, proving that the methodologies elaborated in the present work can effectively be used for the preparation of water-soluble polymeric Ir(III) complexes focusing on optical applications in aqueous environments.

**Figure 4:**
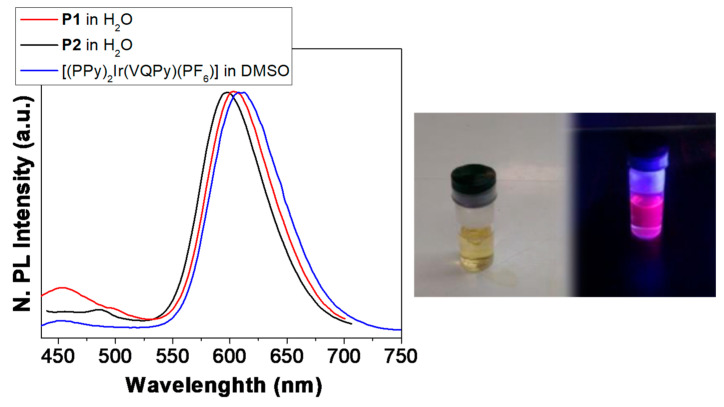
Photoluminescence spectra of the copolymers **P1** and **P2** in aqueous solution and of the monomer complex [(PPy)_2_Ir(VQPy)(PF_6_)] in DMSO, after excitation at 400 nm. The photographs in the right, show the aqueous solution of **P1** in daylight (left photo) and under UV illumination at 365 nm (right photo).

In addition to the absorption characteristics, the copolymers maintain also the emission characteristics of the Ir(III) complexes in aqueous environment, as seen in Figure 4 where the normalized photoluminescence spectra of products **P1** and **P2** in water and of the initial vinylic [(PPy)_2_Ir(VQPy)(PF_6_)] complex in DMSO are compared, upon excitation at 400 nm. Under these conditions an emission peak at 610 nm is clearly detectable. This characteristic band is assigned to the strong singlet and triplet metal-to-ligand charge transfer (MLCT) transition of the Ir(III) complex. As a result, the aqueous solutions of products **P1** and **P2** emit red light under UV illumination as shown in the right part of Figure 4.

**Figure 5:**
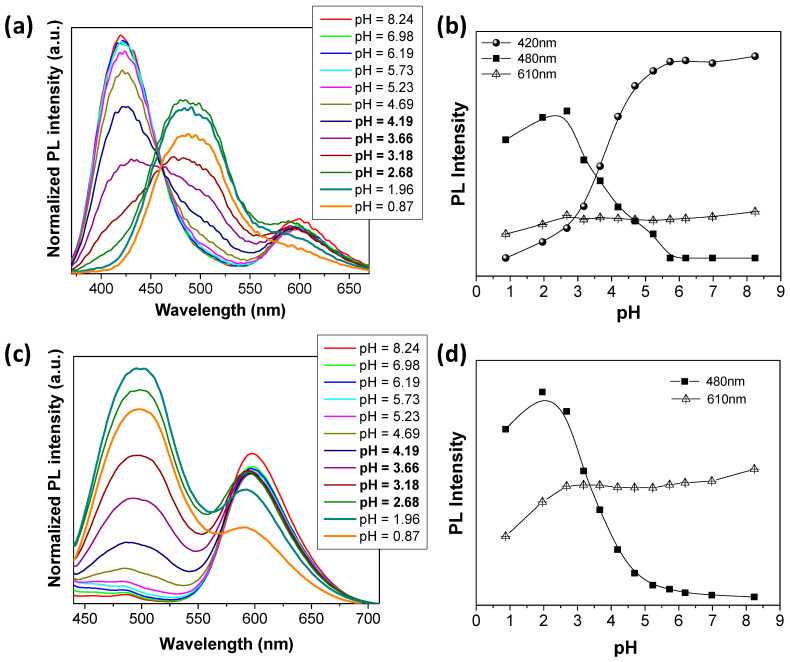
pH-dependence emission spectra after excitation at (**a**) 350 nm and (**c**) 400 nm for copolymer P(DMAM-co-VQPy-co-[(PPy)_2_Ir(VQPy)(PF_6_)]) **P2**. (**b**,**d**) provide the pH-dependence of the emission intensity of the unprotonated VQPy (420 nm), protonated VQPy (480 nm) and of the Ir complexed VQPy (610 nm) species after excitation at (**b**) 350 nm and at (**d**) 400 nm.

Though no detailed study was performed, we have seen that this red color is practically unaffected by pH changes in the case of product **P1**. In contrast, in the case of product **P2**, containing both Ir(III)-complexed and free VQPy units, a very interesting pH-controlled photoluminescence behavior was observed. To demonstrate this behavior, we recorded the photoluminescence spectra of terpolymer **P2** in buffer solutions covering the pH range 2–8, upon excitation at 350 nm and 400 nm (Figure 5). As seen in Figure 5, the red emission peak of the Ir(III) complex at 610 nm is rather constant, regardless of pH. On the other hand, it is known that the emission properties of quinoline-based products are controlled by the acidity of the environment, as a consequence of the weak basic character of quinoline units [52,53,62]. More specifically, quinoline-based polymers can respond to the acidity of their solution due to the weak basic character of quinoline. The protonation of the quinoline unit when incorporated in a polymer, results into a small red-shift of the polymer’s absorption spectrum. The emission spectrum, on the other hand, is more evidently red-shifted (even up to 100 nm). Moreover, the emission peak of the protonated quinoline-based polymers is much broader and significantly weaker. The large shift of the emission maximum to higher wavelengths in acidic media is an indication for the formation of aggregates originating from intra- or intermolecular interactions attributed to acid-based interactions of the quinoline group leading to excimer formation [52]. Actually, quinoline homopolymers or quinoline-rich copolymers show significantly quenched emission of the protonated form of the quinoline unit [62]. On the other hand, the green emission of the protonated quinoline unit can be clearly observed when luminescence quenching is suppressed thus, not allowing the formation of excimers, e.g., when fixed onto carbon nanotubes [67] or when low fractions of quinoline units are randomly fixed onto cross-linked polymeric nanoparticles [54]. Through this latter approach pH-sensing applications have been demonstrated for water-soluble quinoline-based copolymers having low quinoline content. These quinoline-labeled water-soluble polymers exhibit sensitive pH-responsive photoluminescent properties, showing blue to green color gradual changes under UV irradiation upon decreasing pH [51,55].

**Figure 6:**
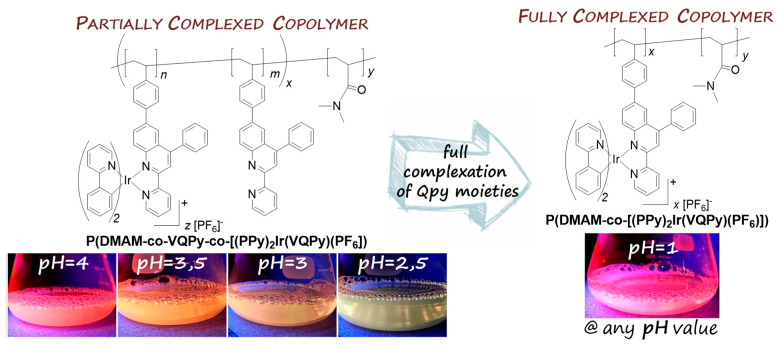
Demonstration of the pH induced color changes of aqueous solutions of copolymer P(DMAM-co-VQPy-co-[(PPy)_2_Ir(VQPy)(PF_6_)]) **P2** and of the persistent red color of the **P2** copolymer bearing only Ir(III)-complexed VQPy moieties. All solutions are shown under UV illumination at 365 nm.

Thus herein, in agreement with previous observations by our group for similar water-soluble copolymers bearing free quinoline-based units [51,55,56,57,58], an emission peak at ~420 nm is observed in Figure 5a upon excitation of aqueous **P2** solutions at 350 nm at high pH, attributed to the unprotonated form of VQPy. Apparently, for our system this blue emission does not perturb the observed color of the solution, which remains red from pH = 8 down to pH = 4.5 (Figure 6, left), due to the emitted color of the Ir(III) complex. Upon further pH decrease, free VQPy units turn to their protonated form and an emission band, reflecting a blue-green color, centered at ~480 nm is seen, while the emission band at 420 nm gradually disappears. Apart from the band at 610 nm, the evolution of both bands is clearly observable when the system is excited at 350 nm (Figure 5a), whereas only the evolution of the green emission of VQPy is monitored upon excitation at 400 nm (Figure 5b), since the absorption of this unit at 400 nm is negligible. As a result of the combination of this green emission from VQPy with the red emission of the Ir(III) complex, a gradual color change of the solution as pH decreases below the value pH = 4.5 is seen, to finally attain a yellow-green color at pH = 2.5 (Figure 6).

The clear color changes shown in Figure 6 support that terpolymer **P2** has the potential for applications as a luminescent indicator/sensor nanomaterial for the acidic pH region. The observed color changes are due to the terpolymer’s exact chemical structure in which the coexistence of free VQPy and iridium-complexed VQPy moieties allows the protonation of the free VQPy at acidic pH environments, therefore leading to PL emission changes and visibly clear color changes of the polymer metallocomplex solutions at the various pH values. This combination of the emission of free VQPy units and Ir(III) complexes, demonstrates that such terpolymer libraries with adequately adjusted compositions may permit the fine tuning of this pH-responsive emission in the acidic region.

Though such a detailed study will be the subject of a future work, to test the basic idea, i.e., that the color changes are the result of the terpolymer chemical structure of **P2**, we proceeded to the full complexation of the remaining free VQPy units with an excess of the chloro-bridge dinuclear [(PPy)_2_IrCl_2_]_2_ complex to obtain the copolymer **P2** (*m* = 0) (Scheme 2 **P2** in which *m* = 0). In this case, no free VQPy are available anymore and the red color persists within the whole pH region, even if pH is as low as 1 (Figure 6, right).

## 4. Conclusions

The complexation of Ir(III) with a vinylic unit containing quinoline-pyridine groups (VQPy) has been used in the present work for the preparation of water-soluble polymeric Ir(III) complex nanomaterials. Two synthetic methodologies have been elaborated, namely pre- or post-polymerization complexation of Ir(III) with VQPy.

Here, we mostly focused on the optical properties, especially emission, of the novel copolymers in aqueous solution. In fact, it is shown that the post-polymerization complexation methodology may lead to terpolymers containing both free and Ir(III)-complexed VQPy units. In this case, the MLCT emission of the Ir(III) complex is combined with the pH-responsive emission of free VQPy units, leading to characteristic pH-responsive color changes under UV illumination in the acidic pH region. Apart from the preparation of pH-responsive luminescent sensory nanomaterials, the present materials could also prove useful for other applications where water-solubility of luminescent Ir(III) complexes is needed, for example cell imaging applications. Moreover, the methodologies elaborated in the present work offer two facile and easily adaptable routes for the incorporation of Ir(III) complexes in more complicated polymeric architectures [68,69].

## Data Availability

Data presented in this article are available on request from the corresponding author.

## References

[1] Reddy M.L.P., Bejoymohandas K.S. (2016). Evolution of 2,3-bipyridine class of cyclometalating ligands as efficient phosphorescent iridium(III) emitters for applications inorganic light emitting diodes. J. Photochem. Photobiol. C Photochem. Rev..

[2] Whittell G.R., Manners I. (2007). Metallopolymers: New Multifunctional Materials. Adv. Mater..

[3] Yersin H. (2008). Highly Efficient OLEDs with Phosphorescent Materials.

[4] Lo K.K., Li S.P. (2014). Utilization of the photophysical and photochemical properties of phosphorescent transition metal complexes in the development of photofunctional cellular sensors, imaging reagents, and cytotoxic agents. RSC Adv..

[5] Polosan S., Radu I. (2013). Mechanisms of the charge transfer in IrQ(ppy)_2_-5Cl dual-emitter compound. J. Nanosci. Nanotechnol..

[6] Shang X., Liu Y., Qu X., Wu Z. (2013). Theoretical study on electronic structures and optical properties of blue phosphorescent iridium(III) complexes witn C^N and N^N ligands. J. Lumin..

[7] Martir D.R., Zysman-Colman E. (2018). Supramolecular iridium(III) assemblies. Coord. Chem. Rev..

[8] Kozhevnikov V.N., Zheng Y., Clough M., Al-Attar H.A., Griffiths G.C., Abdullah K., Raisys S., Jankus V., Bryce M.R., Monkman A.P. (2013). Cyclometalated iridium(III) complexes for high-efficiency solution-processable blue PhOLEDs. Chem. Mater..

[9] Park G., Seo J., Kim Y., Kim Y.S. (2007). Iridium(III) complexes with 6-pentafluorophenyl-2,4-diphenylquinolines for red OLEDs. Mol. Cryst. Liq. Cryst..

[10] Lai W., Levell J., Jackson A., Lo S., Bernhardt P., Samuel I., Burn P. (2010). A phosphorescent poly(dendrimer) containing iridium(III) complexes: Synthesis and light-emitting properties. Macromolecules.

[11] Zysman-Colman E. (2017). Iridium(III) in Optoelectronic and Photonics Applications.

[12] Ma Υ., Liu S., Yang H., Wu Y., Yang C., Liu X., Zhao Q., Wu H., Liang J., Li F. (2011). Water-Soluble phosphorescent iridium(III) complexes as multicolor probes for imaging of homocysteine and cysteine in living cells. J. Mater. Chem..

[13] Shiu H.-Y., Chong H.-C., Leung Y.-C., Zu T., Che C.-M. (2014). Phosphorescent proteins for bio-imaging and site selective bio-conjugation of peptides and proteins with luminescent cyclometalated iridium(III) complexes. Chem. Commun..

[14] Caporale C., Massi M. (2018). Cyclometalated iridium(III) complexes for life science. Coord. Chem. Rev..

[15] Gao H., Li Z., Zhao Y., Qi H., Zhang C. (2017). Aldehyde bearing bis-cyclometalated Ir(III) complex as selective photoluminescence turn-on probe for imaging intracellular homocysteine. Sens. Actuators B..

[16] Yip A.M.-H., Lo K.K.-W. (2018). Luminescent rhenium(I), ruthenium(II), and iridium(III) polypyridine complexes containing a poly(ethylene glycol) pendant or bioorthogonal reaction group as biological probes and photocytotoxic agents. Coord. Chem. Rev..

[17] Zhai T.L., Wang C.C., Cui L.L., Du J., Zhou Z.G., Yang H., Yang S.P. (2020). Hollow Bimetallic Complex Nanoparticles for Trimodality Imaging and Photodynamic Therapy In Vivo. ACS Appl. Mater. Interfaces.

[18] Gupta A., Prasad P., Shalini G., Sasmal P.K. (2020). Simultaneous Ultrasensitive Detection and Elimination of Drug-Resistant Bacteria by Cyclometalated Iridium(III) Complexes. ACS Appl. Mater. Interfaces.

[19] Lalevee J., Tehfe M., Dumur F., Gigmes D., Blanchard N., Morlet-Savary F., Fouassier J. (2012). Iridium photocatalysts in free radical photopolymerization under visible lights. ACS Macro Lett..

[20] Lalevee J., Dumur F., Mayer C., Gigmes D., Nasr G., Tehfe M., Telitel S., Morlet-Savary F., Graff B., Fouassier J. (2012). Photopolymerization of N-Vinylcarbazole using visible-light harvesting iridium complexes as photoinitiators. Macromolecules.

[21] Sun Z., Liu Y., Chen J., Huang C., Tu T. (2015). Robust Iridium Coordination Polymers: Highly Selective, Efficient, and Recyclable Catalysts for Oxidative Conversion of Glycerol to Potassium Lactate with Dihydrogen Liberation. ACS Catal..

[22] Ainembabazi D., Wang K., Finn M., Ridenour J., Voutchkova-Kostal A. (2020). Efficient transfer hydrogenation of carbonate salts from glycerol using water-soluble iridium N-heterocyclic carbene catalysts. Green Chem..

[23] van Lier R.C.W., de Bruijn A.D., Roelfes G. (2021). A Water-Soluble Iridium Photocatalyst for Chemical Modification of Dehydroalanines in Peptides and Proteins. Chem. Eur. J..

[24] Schaferling M. (2012). The art of fluorescence imaging with chemical sensors. Angew. Rev..

[25] Tian N., Lenkeit D., Pelz S., Fischer L., Escudero D., Scchiewek R., Klink D., Schmitz O., Gonzalez L., Schaferling M. (2010). Structure-property relationship of red- and green- emmiting iridium (III) complexes with respect to their temperature and oxygen sensitivity. Eur. J. Inorg. Chem..

[26] Chan D.S.-H., Fu W.-C., Wang M., Liu L.-J., Leung C.-H., Ma D.-L. (2013). A highly selective and non-reaction based chemosensor for the detection of Hg^2+^ ions using a luminescent iridium(III) complex. PLoS ONE.

[27] Mei J., Leung N.L.C., Kwok R.T.K., Lam J.W.Y., Tang Z.B. (2015). Aggregation-Induced Emission: Together We Shine, United We Soar!. Chem. Rev..

[28] Zhang Q., Zhou M. (2015). A profluorescent ratiometric probe for intracellular pH imaging. Talanta.

[29] Tobita S., Yoshihara T. (2016). Intracellular and in vivo oxygen sensing using phosphorescent iridium(III) complexes. Curr. Opin. Chem. Biol..

[30] Liu S., Wei L., Guo S., Jiang J., Zhang P., Han J., Ma Y., Zhao Q. (2018). Anionic iridium(III) complexes and their conjugated polymer soft salts for time-resolved luminescent detection of intracellular oxygen levels. Sens. Actuators B.

[31] You Y., Cho S., Nam W. (2014). Cyclometalated Iridium(III) Complexes for Phosphorescence Sensing of Biological Metal Ions. Inorg. Chem..

[32] Smith R.A., Stokes E.C., Langdon-Jones E.E., Platts J.A., Kariuki B.M., Hallett A.J., Pope S.J.A. (2013). Cyclometalated cinchophen ligands on iridium(III): Towards water-soluble complexes with visible luminescence. Dalton Trans..

[33] Li M.-J., Jiao P., Lin M., He W., Chen G.-N., Chen X. (2011). High electrochemiluminescence of a new water-soluble iridium(III) complex for determination of antibiotics. Analyst.

[34] Jiang W., Gao Y., Sun Y., Ding F., Xu Y., Bian Z., Li F., Bian J., Huang C. (2010). Zwitterionic Iridium Complexes: Synthesis, Luminescent Properties, and Their Application in Cell Imaging. Inorg. Chem..

[35] Yu L., Huang Z., Liu Y., Zhou M. (2012). Photophysics, electrochemistry and electrochemiluminescence of water-soluble biscyclometalated iridium (III) complexes. J. Organomet. Chem..

[36] Fan Y., Zhao J., Yan Q., Chen P.R., Zhao D. (2014). Water-Soluble Triscyclometalated Organoiridium Complex: Phosphorescent Nanoparticle Formation, Nonlinear Optics, and Application for Cell Imaging. ACS Appl. Mater. Interfaces.

[37] Solomatina A.I., Su S.-H., Lukina M.M., Dudenkova V.V., Shcheslavskiy V.I., Wu C.-H., Chelushkin P.S., Chou P.-T., Koshevoy I.O., Tunik S.P. (2018). Water-soluble cyclometalated platinum(II) and iridium(III) complexes: Synthesis, tuning of the photophysical properties, and in vitro and in vivo phosphorescence lifetime imaging. RSC Adv..

[38] Erten-Ela S., Ocakoglu K. (2014). Iridium dimer complex for dye sensitized solar cells using electrolyte combinations with different ionic liquids. Mater. Sci. Semicond..

[39] Jaina N., Alamb P., Laskar I.R., Panwar J. (2015). Aggregation Induced Phosphorescence’ Active Iridium(III) Complexes for Integrated Sensing and Inhibition of Bacteria in Aqueous Solution. RSC Adv..

[40] Scarpelli F., Ionescu A., Ricciardi L., Plastina P., Aiello I., La Deda M., Crispini A., Ghedini M., Godbert N. (2016). A Novel Route Towards Water-Soluble Luminescent Iridium(III) Complexes via a Hydroxy-bridged Dinuclear Precursor. Dalton Trans..

[41] Zanarini S., Rampazzo E., Bonacchi S., Juris R., Marcaccio M., Montalti M., Paolucci F., Prodi L. (2009). Iridium Doped Silica-PEG Nanoparticles: Enabling Electrochemiluminescence of Neutral Complexes in Aqueous Media. J. Am. Chem. Soc..

[42] Ma Y., Liu S., Yang H., Wu Y., Sun H., Wang J., Zhao Q., Li F., Huang W. (2013). A water-soluble polymer for time-resolved assay and bioimaging of cysteine/homocysteine. J. Mater. Chem. B.

[43] Maggioni D., Galli M., D’Alfonso L., Inverso D., Dozzi M.V., Sironi L., Iannacone M., Collini M., Ferruti P., Ranucci E. (2015). A Luminescent Poly(amidoamine)−Iridium Complex as a New Singlet-Oxygen Sensitizer for Photodynamic Therapy. Inorg. Chem..

[44] Sun P., Lu X., Fan Q., Zhang Z., Song W., Li B., Huang L., Peng J., Huang W. (2011). Water-Soluble Iridium(III)-Containing Conjugated Polyelectrolytes with Weakened Energy Transfer Properties for Multicolor Protein Sensing Applications. Macromolecules.

[45] Metera K.L., Hänni K.D., Zhou G., Nayak M.K., Bazzi H.S., Juncker D., Sleiman H.F. (2012). Luminescent Iridium(III)-Containing Block Copolymers: Self-Assembly into Biotin-Labeled Micelles for Biodetection Assays. ACS Macro Lett..

[46] Liu S., Qiao W., Cao G., Chen Y., Ma Y., Huang Y., Liu X., Xu W., Zhao Q., Huang W. (2013). Smart Poly(N-isopropylacrylamide) Containing Iridium(III) Complexes as Water-Soluble Phosphorescent Probe for Sensing and Bioimaging of Homocysteine and Cysteine. Macromol. Rapid Commun..

[47] Hou H., Sun P., Fan Q., Lu X., Xue C., Zhang Y., Tian S., Huang W. (2015). Synthesis of Water-Soluble Iridium (III)-Containing Nanoparticles for Biological Applications. J. Chem..

[48] Itsuno S., Hashimoto Y., Haraguchi N. (2014). Synthesis of chiral iridium complexes immobilized on amphiphilic polymers and their application to asymmetric catalysis. J. Polym. Sci. Part A Polym. Chem..

[49] Sun P., Wang G., Hou H., Yuan P., Deng W., Wang C., Lu X., Fan Q., Huang W. (2017). A water-soluble phosphorescent conjugated polymer brush for tumor-targeted photodynamic therapy. Polym. Chem..

[50] Chen Z., Meng X., Xie M., Shi Y., Zou L., Guo S., Jiang J., Liu S., Zhao Q. (2020). A self-calibrating phosphorescent polymeric probe for measuring pH fluctuations in subcellular organelles and the zebrafish digestive tract. J. Mater. Chem. C.

[51] Kalogianni A., Pefkianakis E., Stefopoulos A., Bokias G., Kallitsis J.K. (2010). pH-responsive photoluminescence properties of a water-soluble copolymer containing quinoline groups in aqueous solution. J. Polym. Sci. Part B Polym. Phys..

[52] Lu L., Jenekhe S. (2001). Poly(vinyl diphenylquinoline):  A New pH-Tunable Light-Emitting and Charge-Transport Polymer Synthesized by a Simple Modification of Polystyrene. Macromolecules.

[53] Zhang X., Shetty A., Jenekhe S. (1999). Electroluminescence and Photophysical Properties of Polyquinolines. Macromolecules.

[54] Moutsiopoulou A., Andreopoulou A.K., Lainioti G., Bokias G., Voyiatzis G., Kallitsis J.K. (2015). Quinoline-functionalized cross-linked poly(vinyl acetate) andpoly(vinyl alcohol) nanoparticles as potential pH-responsive luminescent sensors. Sens. Actuators B.

[55] Thivaios I., Kakogianni S., Bokias G. (2016). A library of quinoline-labeled water-soluble copolymers with pH-tunable fluorescence response in the acidic pH region. Macromolecules.

[56] Kougia E., Tselepi M., Vasilopoulos G., Lainioti G.C., Koromilas N.D., Druvari D., Bokias G., Vantarakis A., Kallitsis J.K. (2015). Evaluation of antimicrobial efficiency of new polymers comprised by covalently attached and/or electrostatically bound bacteriostatic species, based on quaternary ammonium compounds. Molecules.

[57] Thivaios I., Koukoumtzis V., Kallitsis J.K., Bokias G. (2016). Quinoline-labeled poly(N-isopropylacrylamide): A selective polymeric luminescent sensor of cationic surfactants. Sens. Actuators B.

[58] Iatridi Z., Vamvakidis K., Tsougos I., Vassiou K., Dendrinou-Samara C., Bokias G. (2016). Multifunctional Polymeric Platform of Magnetic Ferrite Colloidal Superparticles for Luminescence, Imaging, and Hyperthermia Applications. ACS Appl. Mater. Interfaces.

[59] Gioti M., Tselekidou D., Panagiotidis L., Kyriazopoulos V., Simitzi K., Andreopoulou A.K., Kalitsis J.K., Gravalidis C., Logothetidis S. (2021). Optical characterization of organic light-emitting diodes with selective red emission. Mater. Today Proc..

[60] Andrikopoulos K., Anastasopoulos C., Kallitsis J.K., Andreopoulou A.K. (2020). Bis-Tridendate Ir(III) Polymer-Metallocomplexes: Hybrid, Main-Chain Polymer Phosphors for Orange—Red Light Emission. Polymers.

[61] Margariti A., Papakonstantinou V.D., Stamatakis G., Demopoulos C.A., Schnakenburg G., Andreopoulou A.K., Giannopoulos P., Kallitsis J.K., Philippopoulos A.I. (2020). Substituted pyridine-quinoline ligands as building blocks for neutral rhodium(III) complexes. Synthesis, structural characterization studies and anti-platelet activity towards the Platelet-Activating Factor (PAF). Polyhedron.

[62] Economopoulos S., Andreopoulou A.K., Gregoriou V., Kallitsis J. (2005). Synthesis and Optical Properties of New End-Functionalized Polyquinolines. Chem. Mater..

[63] Kappaun S., Eder S., Sax S., Mereiter K., List E., Slugove C. (2007). Organoiridium Quinolinolate Complexes: Synthesis, Structures, Thermal Stabilities and Photophysical Properties. Eur. J. Inorg. Chem..

[64] Donato L., McCusker C., Castellano F., Colman E. (2013). Mono- and dinuclera cationic iridium(II) complexes bearing a 2,5-dipyridylpyrazine (2,5-bpp) ligand. Inorg. Chem..

[65] Park G.Y., Kim Y.S. (2008). Synthesis and photophysical study of iridium(III) complex with 6-pentafluorophenyl-2,4-diphenylquinolines. Colloids Surf. A Physicochem. Eng. Asp..

[66] Lamansky S., Djurovich P., Murphy D., Abdel-Razzaq F., Lee H.-E., Adachi C., Burrows P.E., Forrest S.R., Thompson M.E. (2001). Highly phosphorescent bis-cyclometalated iridium complexes: Synthesis, photophysical characterization, and use in organic light emitting diodes. J. Am. Chem. Soc..

[67] Stefopoulos A.A., Kourkouli S.N., Economopoulos S., Ravani F., Andreopoulou A., Siokou A., Papaggelis K., Kallitsis J.K. (2010). Polymer and hybrid electron accepting materials based on a semiconducting perfluorophenylquinoline. Macromolecules.

[68] Pefkianakis E., Tzanetos N., Kallitsis J. (2008). Synthesis and characterization of a Novel Vinyl-2,2′-bipyridine monomer and its homopolymeric/copolymeric metal complexes. Chem. Mater..

[69] Aivali S., Kakogianni S., Anastasopoulos C., Andreopoulou A.K., Kallitsis J.K. (2019). Copolymers and Hybrids Based on Carbazole Derivatives and Their Nanomorphology Investigation. Nanomaterials.

